# The first step is recognizing there is a problem: a methodology for adjusting for variability in disease severity when estimating clinician performance

**DOI:** 10.1186/s12874-022-01543-7

**Published:** 2022-03-16

**Authors:** Meagan Bechel, Adam R. Pah, Stephen D. Persell, Curtis H. Weiss, Luís A. Nunes Amaral

**Affiliations:** 1 Feinberg School of Medicine, Northwestern University, Chicago, IL USA; 2 Northwestern Institute on Complex Systems, Northwestern University, 2145 Sheridan Road (Room E136), Evanston, IL 60208 USA; 3 Kellogg School of Management, Northwestern University, Evanston, IL USA; 4 Division of General Internal Medicine and Geriatrics, Feinberg School of Medicine, Northwestern University, Chicago, IL USA; 5 Center for Primary Care Innovation, Institute for Public Health and Medicine, Feinberg School of Medicine, Northwestern University, Chicago, IL USA; 6 Division of Pulmonary, Critical Care, Allergy, and Immunology, NorthShore University HealthSystem, 1001 University Place, Suite 162, Evanston, IL 60201 USA; 7 Department of Chemical and Biological Engineering, Northwestern University, Evanston, IL USA; 8 Department of Physics and Astronomy, Northwestern University, Evanston, IL USA

**Keywords:** Clinical medicine, Performance measure, Data science, Social network analysis, Critical care

## Abstract

**Background:**

Adoption of innovations in the field of medicine is frequently hindered by a failure to recognize the condition targeted by the innovation. This is particularly true in cases where recognition requires integration of patient information from different sources, or where disease presentation can be heterogeneous and the recognition step may be easier for some patients than for others.

**Methods:**

We propose a general data-driven metric for clinician recognition that accounts for the variability in patient disease severity and for institutional standards. As a case study, we evaluate the ventilatory management of 362 patients with acute respiratory distress syndrome (ARDS) at a large academic hospital, because clinician recognition of ARDS has been identified as a major barrier to adoption to evidence-based ventilatory management. We calculate our metric for the 48 critical care physicians caring for these patients and examine the relationships between differences in ARDS recognition performance from overall institutional levels and provider characteristics such as demographics, social network position, and self-reported barriers and opinions.

**Results:**

Our metric was found to be robust to patient characteristics previously demonstrated to affect ARDS recognition, such as disease severity and patient height. Training background was the only factor in this study that showed an association with physician recognition. Pulmonary and critical care medicine (PCCM) training was associated with higher recognition (β = 0.63, 95% confidence interval 0.46–0.80, *p* < 7 × 10^− 5^). Non-PCCM physicians recognized ARDS cases less frequently and expressed greater satisfaction with the ability to get the information needed for making an ARDS diagnosis (*p* < 5 × 10^− 4^), suggesting that lower performing clinicians may be less aware of institutional barriers.

**Conclusions:**

We present a data-driven metric of clinician disease recognition that accounts for variability in patient disease severity and for institutional standards. Using this metric, we identify two unique physician populations with different intervention needs. One population consistently recognizes ARDS and reports barriers vs one does not and reports fewer barriers.

**Supplementary Information:**

The online version contains supplementary material available at 10.1186/s12874-022-01543-7.

## Background

The first step to solving a problem is recognizing that one exists; this principle is widely accepted and contributes to the foundation of both implementation science and quality improvement. In clinical medicine, this principle is applied on multiple levels of care delivery. At the organizational level, healthcare institutions monitor quality of care indicators such as nosocomial infection incidence, readmission rates, and clinician guideline adherence, with the goal of intervening early should an issue develop. Routine data collection and analysis is far more cost-effective than routine interventions on all individuals or units within an institution. In this work, we extend this same logic to the individual clinician level, where recognition of a problem is frequently synonymous with recognition of disease.

At the individual provider level, recognition of disease is the first step in all clinical decision making. No medical treatment is fully benign and therefore, treatment is not indicated without a diagnosis or high suspicion of a specific diagnosis. As a result, recognition of disease becomes a prerequisite to any decision regarding the adoption of a clinical innovation. If a clinician decides that a disease state does not exist in a patient, there is no need for that clinician to consider potential treatment choices for that supposedly non-existent disease state. Thus, disease recognition challenge needs to be addressed before any other barrier to adoption.

However, the relative difficulty of the disease recognition task can vary between patients, making measuring clinician disease recognition challenging. The same condition can present with different clinical signs and/or on a spectrum of severity, with some diagnoses being easier than others. Thus, while simple performance measures of clinician disease recognition – such as proportion of correct diagnoses – are useful for patient outcome assessment, it does not capture the complexity of patient care and has limited utility for comparing individual clinicians at the same institution, the same clinician over time, or untangling an individual clinician’s performance from that of the overall institution. It is the same logic that drives using risk-adjustment when calculating readmission rates; there are many factors that go into whether a patient will be readmitted, only some of which a physician can reasonably intervene on. Readmissions cannot be fully eliminated by the hospital staff, but they can be minimized by certain staff actions and institutional resources and policies.

In this work, we extend this principle to clinician disease recognition and present a data-driven methodology that accounts for variability in patient disease severity. The goal of this recognition measure is not to serve as a patient care quality measure, but instead be a standardized measure for evaluating a physician’s progress towards a patient care goal within the context of the overall performance of the institution. Using this metric, we then examine the relationship between recognition and physician characteristics - such as demographics, social network position, and self-reported barriers – using the case study of ventilator management in patients with acute respiratory distress syndrome.

### Low tidal volume ventilation (LTVV) for acute respiratory distress syndrome (ARDS)

Historically, the adoption of innovation in clinical medicine has been slow [1–4] and the use of LTVV for patients with ARDS is a prime example. ARDS is a syndrome of acute inflammatory lung injury leading to hypoxemic respiratory failure. It is frequently associated with a known risk factor, such as pneumonia or inhalation injury [5], and thus often found in critically-ill and medically complex patients. Prior to the arrival of the COVID-19 pandemic, ARDS affected 10.4% of intensive care unit (ICU) admissions worldwide [5–7]. The current prevalence is likely higher given that SARS-CoV-2 infection has been shown to be a risk factor for ARDS [8]. Pre-pandemic mortality estimates for ARDS ranged from 27 to 45% [6], with additional reports of significant post-recovery morbidity [9, 10].

LTVV is a ventilator management strategy focused on lowering mechanically-delivered volumes and pressures with the goal of preventing ventilator-associated lung injury. It was first trialed in patients with ARDS in the 1998 and multiple subsequent trials have shown that it reduces mortality [11, 12]. LTVV has since become a strongly-recommended practice for patients with ARDS, incorporated into the American Thoracic Society guidelines [12].

**Fig. 1:**
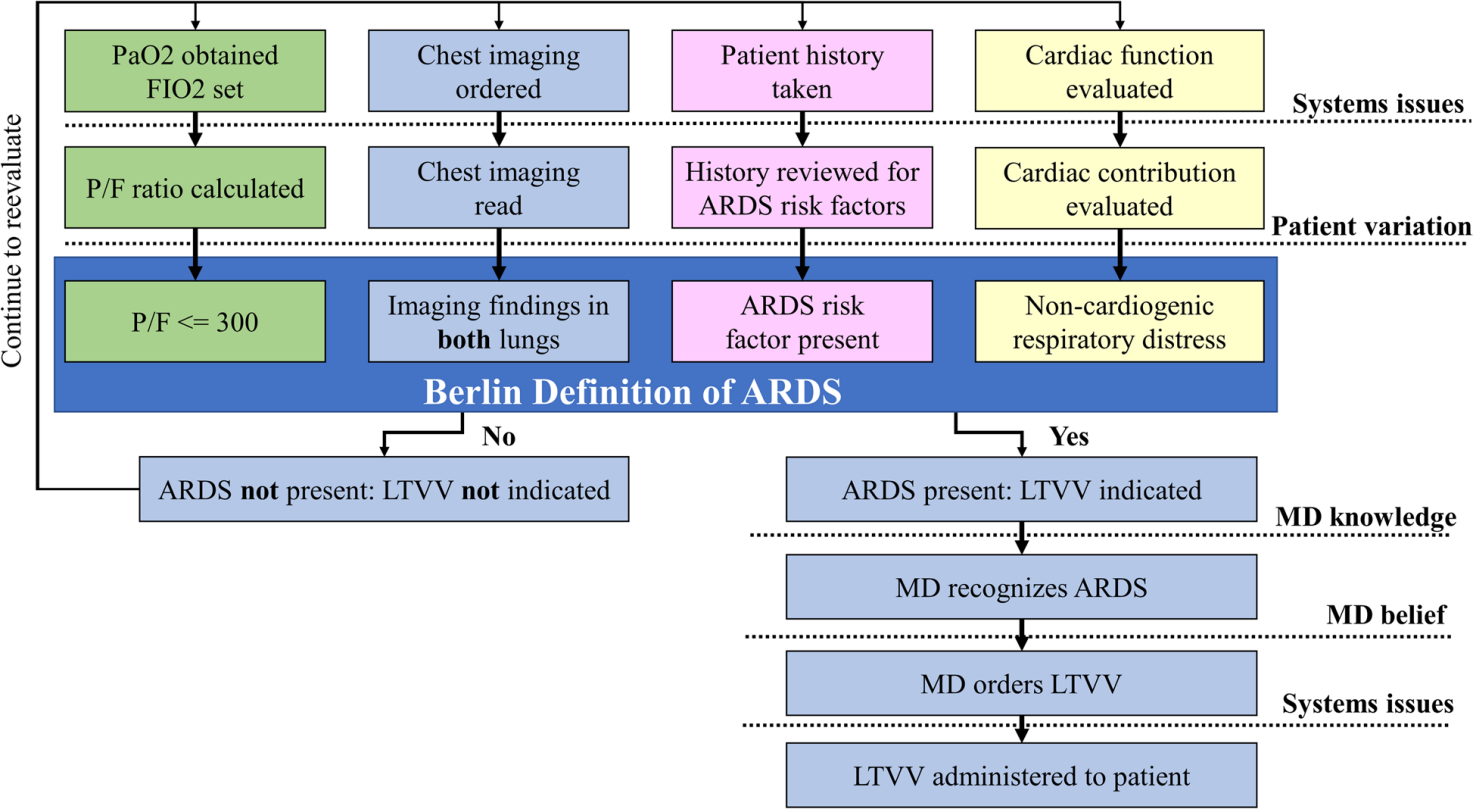
Implementation of LTVV for ARDS. The implementation of LTVV is a multi-step process that starts with ARDS development and recognition. ARDS recognition requires the synthesis of multiple types of clinical information. The standardized tidal volume delivered to a patient, whether it falls within the LTVV range or not, is the end product of clinician decision making. Several potential barriers (dotted lines) may delay or prevent the implementation of LTVV.

Yet, clinical LTVV utilization remains as low as 19% of ARDS patients [7, 13–20]. Research has demonstrated that while physician ‘buy-in’ for LTVV is high, ARDS under-recognition remains a major barrier to LTVV use [7, 17–26]. Furthermore, there is significant evidence that patient characteristics, such as ARDS severity, strongly influence physician recognition of ARDS [7, 15, 17, 19, 21, 27]. ARDS is diagnosed using the Berlin Definition [5], which is a set of clinical criteria that a patient must meet. The Berlin Definition (Fig. 1) requires the synthesis of multiple sources of information, including lab values, ventilator settings, imaging, and patient history. ARDS is an ideal case study for our recognition metric due to the clear need for an increase in recognition, the complexity of its diagnosis, and the variability of its presentation.

## Methods

### Data acquisition

#### Patient data

We have previously described the development and assessment of the ARDS cohort in this study, which included 362 patients who met the Berlin Definition of ARDS at four hospitals in the Chicago region in 2013 [20]. Patient data was obtained from the electronic health records serving the participating hospitals. For this study, we use height and sex to calculate predicted body weight (sex neutral surrogate for height) as well as all tidal volumes (mL/kg) and P_a_O_2_/F_I_O_2_ ratios (mmHg) available during the patient’s disease course. These data were collected as part of an observational study focused on understanding ARDS recognition and management, and implementation of LTVV. The methods and insights gleaned from this work will be incorporated into a larger multicenter study of ARDS recognition and management, and barriers to implementation of LTVV (NIH R01 HL140362).

#### Physician data

We have previously described the survey used in this study, which included the critical care physicians who were identified as caring for the patients in the ARDS cohort described above [26]. The survey included questions on physician attitudes towards LTVV and innovation in general, perceived barriers and facilitators to LTVV use, and professional and social connections with other ICU physicians. Physicians who met cohort inclusion criteria but were missing data points were only excluded from the analyses that requires those missing data points. Data availability is reported in Additional File, Supp Table 1.

### Calculation of ARDS recognition

Our ARDS recognition metric that compares an individual physician’s observed ARDS recognition to that physician’s expected ARDS recognition given their specific patient census. The calculation of each physician’s recognition metric includes only the data generated during that physician’s specific pairing with his/her patients. Due to different data collection procedures at different clinical sites, we were only able to calculate the ARDS recognition metric for the largest site in our previous study [20].

#### Observed recognition

**Fig. 2:**
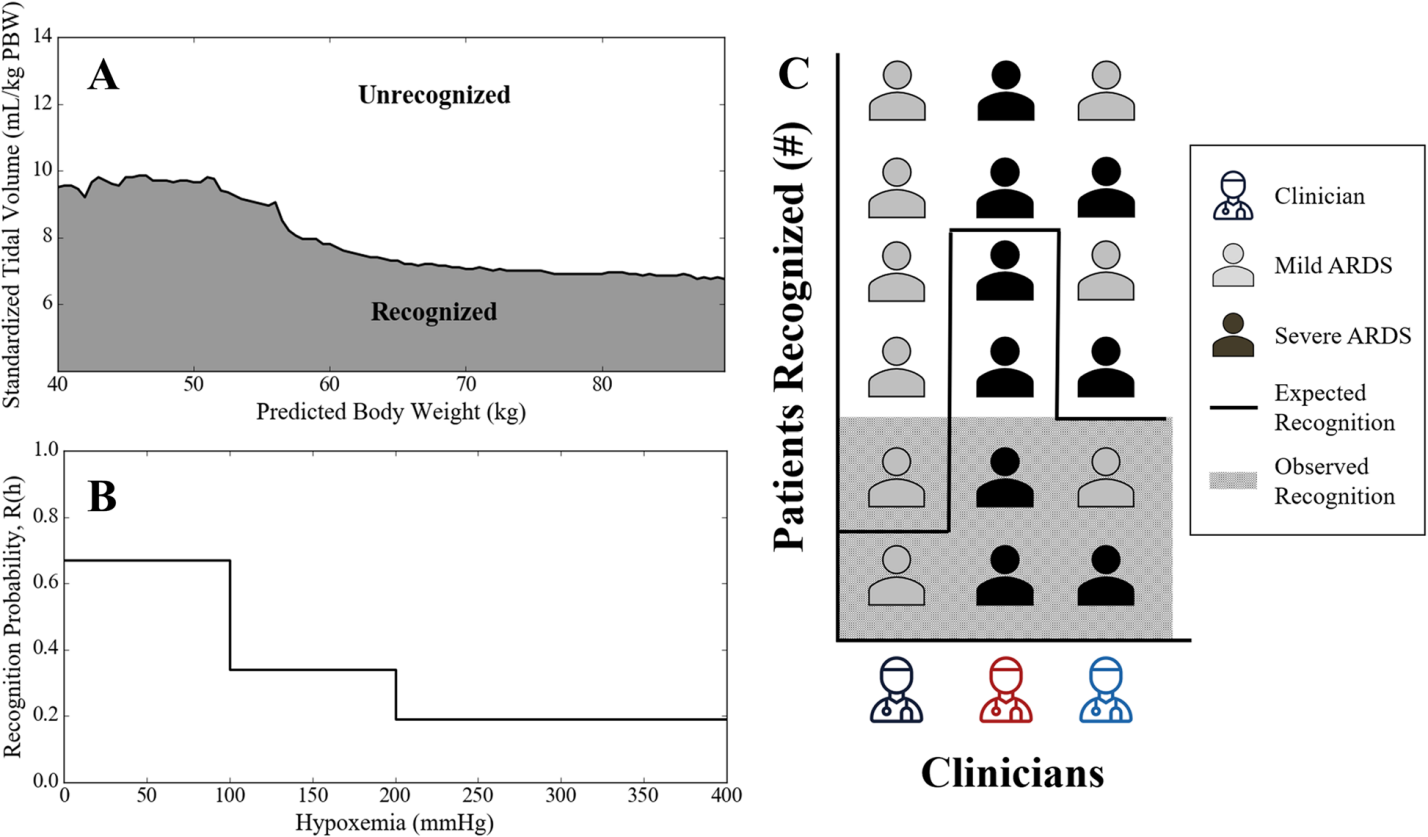
Components of the ARDS recognition metric. A) Observed Recognition: we designate an ARDS patient as recognized if their standardized tidal volume falls below the recognition line for their predicted body weight. B) Expected Recognition: we use a stepwise function relating hypoxemia and recognition probability in eq. 1 to calculate an expected baseline recognition rate for each physician. C) Recognition Metric: we compare the observed and expected recognition for each physician to account for patient presentation variability.

For each patient in a physician’s census, we assign a label of “recognized” or “not recognized.” This label is inferred from the standardized tidal volume selected by the clinician to be delivered to the patient. This inference is based on our previously developed model of physician recognition of ARDS. Previously, we quantified the impact of patient characteristics on physician recognition of ARDS and subsequent LTVV delivery, by comparing physician behavior with ARDS patients to physician behavior with a novel hypoxemic ‘control’ cohort [27]. We found that the largest confounding characteristics in both ARDS and control cohorts was patient height (reported as the sex-neutral ‘predicted body weight’). We developed a model that accounts for this by dividing the predicted body weight (PBW) vs standardized tidal volume (mL/kg PBW) space into “recognized” and “not recognized” regions (Fig. 2A). Patients in the “recognized” region experience physician behavior more similar to that exhibited with ARDS patients as compared to physician behavior seen with the control patients. In this work, we map each physician’s patient census to this space and infer their observed individual recognition, N_obs_, as the number of their patients falling within the “recognized” region.

#### Expected recognition

From a patient outcomes perspective, a physician’s expected recognition would be 100% of the ARDS patients they see. However, the goal of our recognition metric is to measure a physician’s progress relative to another physician, their progress over time, and/or to an institution’s past average; it is not intended to replace overall missed diagnosis rates. Thus, we use the current group average of all physicians within the same institution as a physician’s expected recognition performance. The idea is that by identifying both high and low performers, the institution will be better able to learn which physicians should be targeted in order to improve overall institutional performance.

In order to accomplish this, we must account for the severity of ARDS (measured by the hypoxemia categories set forth in the Berlin definition) because we and others have previously demonstrated that the severity of ARDS has an impact on a physician’s ability to recognize ARDS, with sicker patients being easier to recognize [7, 22]. To establish a baseline expected recognition rate for each physician that accounts for this influence, we used the following equation:

1 $${N}_{exp}\left(\left\{{h}_1,{h}_2,\dots {h}_{N_j}\right\}\right)= floor\left[{\sum}_{i=1}^{N_j}R\left({h}_i\right)\right]$$

where:

N_exp_: expected number of patients to be recognized.

h_i_: hypoxemia severity category (mild, moderate, or severe) [5] of patient i.

R(h_i_): institutional level recognition rates of mild, moderate, or severe patients [27] (Fig. 2B).

N_j_: number of patients cared for by physician j.

The recognition rates, R(h_i_), in Eq. 1 are for the whole ARDS cohort by hypoxemia severity, which we estimated in prior work via mixture model as 22% for mild hypoxemia (P_a_O_2_/F_I_O_2_ in range 200-300 mmHg), 34% for moderate hypoxemia (P_a_O_2_/F_I_O_2_ in range 100-200 mmHg), and 67% for severe (P_a_O_2_/F_I_O_2_ < 100 mmHg) [27]. Expected recognition is rounded down to the nearest whole patient to account for the binary nature of ARDS diagnosis.

#### ARDS recognition metric

Our ARDS recognition metric *R* (Fig. 2C) compares the cumulative probabilities of the observed and expected recognition scenarios

2 $$R=P\left(\le {N}_{obs}\right)-P\left(\le {N}_{exp}\right).$$

To calculate *R*, consider a physician with a patient census {h_1_, …, h_Nj_}, and that for each patient *i* in the census there is an institutional level recognition rate, *R*(h_i_), appropriate for the patient’s hypoxemia severity (Fig. 2B). The expected number of recognized patients for that physician’s census is.

3 $$N_{\exp}=N_{j}\;\left(f_{\mathrm{mild}}\;\mathrm R\left(h_{\mathrm{mild}}\right)+f_{\mathrm{interm}}\;\mathrm R\left(h_{\mathrm{interm}}\right)+f_{\mathrm{severe}}\;\mathrm R\left(h_{\mathrm{severe}}\right)\right),$$

where the three subscripts refer to mild, intermediate, and severe hypoxemia, and *f* is the fraction of patients in the census with a given hypoxemia severity. Since the number of patients in a physician’s census may not be large and because N_exp_ is not necessarily an integer, calculating *P*(≤*N*_*obs*_) or *P*(≤*N*_*exp*_) is more easily done by simulation then by enumeration. Thus, we generated 1000 sequences of recognized/not-recognized outcomes for the physician’s patient census according to the recognition probability of each patient’s hypoxemia severity (see Additional File, Supplemental Methods, Probability Density Function Distribution Generation). This process enabled us to estimate the probability of each number between 0 and N_j_ of recognized patients for each physician. By using the cumulative probability (Eq. 2), we ensure that physicians recognizing more patients than expected are assigned positive performance values, while physicians recognizing less patients are assigned negative values. Physicians performing at the expected level for their peer group are rated at 0.

### ARDS recognition metric covariates

#### Metric robustness evaluation

We used univariable ordinary least squares (OLS) regression to assess the robustness of our recognition metric. We evaluated whether our metric showed any correlation with key variables including predicted body weight, hypoxemia (lowest P_a_O_2_/F_I_O_2_), total number of patients treated, and mortality proportion within each physician’s census. For predicted body weight, we used summary statistics of the physician’s patient census (mean, median, proportions in the central, single standard deviation, and second standard deviation ranges) and for hypoxemia, we used the proportion of the patient population with severe hypoxemia (P_a_O_2_/F_I_O_2_  100).

#### Physician characteristics

We sought to evaluate the relative associations between physician recognition of ARDS and physician characteristics that have been previously shown to have an impact on clinical decision-making and use of evidence-based practices: physician demographics [28], social network position [29–39], and attitude survey responses. For demographic variables and social network attributes, we used a feedforward OLS regression approach with our recognition metric as the dependent variable and physician characteristics as independent variables. Demographic univariable analysis was performed first, as demographic characteristics have been previously shown to affect network connections [40–42]. Demographic variables included: training background (specialty), age, sex, and year of training completion (ordinal and before/after ARDSNet LTVV trial [11]).

Next, we constructed four different social networks (patient contact [43], advice seeking, friendship, and innovation) and calculated 8 positional metrics for each physician (betweenness, closeness, degree, Katz centrality, k-shell embeddedness, participation, role, and community membership). For detailed descriptions of network construction and each positional metric, see Additional File, Supplemental Methods, Network Construction, and Additional File, Supp Table 2. All centrality characteristics (betweenness, closeness, degree, and Katz) were calculated using the Networkx Python package (v 1.11), except embeddedness which was calculated using custom code [44]. Participation, role, and community membership were calculated using netcarto (v1.15). All positional metrics were normalized for the number of physicians in the network, except community membership, which was treated as a categorical variable. Significant demographic variables were included as a fixed effect in multivariable OLS regressions with positional metrics as an additional independent variable. Each positional metric was evaluated in a separate regression.

For the survey response analysis, we used both an individual question and a collective group approach. Survey questions (non-demographic, non-network) were first filtered for those that showed a maximum range of responses. To examine associations between individual physician survey responses and physician recognition, we used a Kruskal-Wallis H-test to evaluate differences in recognition between categories of survey answer for each question (Python Scipy package v0.18.1). To evaluate for differences in survey response between groups of physicians, we split physicians by the significant demographic or positional metric identified in the prior feedforward regression analysis. We then used a Mann-Whitney U test to assess differences in the responses from these groups to the same filtered question pool (Python Scipy package v0.18.1).

### Sensitivity analyses

All analyses were repeated using two alternative ARDS recognition measures that have been previously used in literature: 1) the proportion of worked shifts during which the physician delivered LTVV and 2) the proportion of patients that a physician cared for who received LTVV at any point during their disease course. For these alternative measures, we used a strict interpretation of LTVV use (defined as ≤6.5 mL/kg PBW) as put forth by the original ARDSNet LTVV trial [11]. These measures do not adjust for the impact of patient height on standardized tidal volume (mL/kg PBW) or the influence of ARDS severity on clinician recognition of ARDS. All regression results using these alternative recognition metrics as the dependent variable were consistent with the regression results when our recognition metric was used.

### Statistical significance

We used α = 0.01 instead of 0.05 to ensure the statistical strength of our findings [45] and applied the Bonferroni correction for multiple hypotheses. There were 140 comparisons where our recognition metric was the dependent variable, thus we set 7 × 10^− 5^ (0.01/140) as the threshold for statistical significance for these analyses. For the survey analysis, there were 20 questions evaluated, resulting in a threshold of 5 × 10^− 4^ (0.01/20).

## Results

### Collected data

This study includes 92 physicians, 74 (80%) of which responded to the survey. Forty-eight physicians (52%) cared for patients in the ARDS cohort during our data collection period and had their ARDS recognition estimated. Of those physicians who responded to the survey and had their ARDS recognition estimated, the training distribution is as follows: 23 pulmonary/critical care, 10 anesthesia/critical care, 8 cardiology, 5 surgical/critical care, and 2 neurology/critical care. Twenty-six (54%) of these physicians were male and most frequently reported age range was 35–44 years (21, 43%). We evaluated 567 physician-patient pairings as some patients had multiple physicians during their disease course (Additional File, Supp Table 3). Moderate ARDS patients were most common with 247 physician-patient pairings (43.6%) and severe ARDS patients were least common with 117 physician-patient pairings (20.6%). Pulmonary/critical care physicians experienced the highest number of patient-physician pairings (287, 50.6%). However, surgical critical care physicians had a higher median contact hours with their ARDS patients as compared to pulmonary critical care physicians (54.2 h vs. 48.0 h). The percentage of patients with severe ARDS was relatively consistent across the different physician groups, ranging from 19.2% for pulmonary/critical care to 26.3% for cardiology (Additional File, Supp Table 3). There were no significant differences in the distribution of ARDS severity seen by physicians in different specialties (Additional File, Supp Fig. 1).

### ARDS recognition metric covariates

#### Metric robustness

Our ARDS recognition metric showed no correlation with PBW, hypoxemia, or mortality proportion (Additional File, Supp Fig. 2).

#### Physician characteristics

**Fig. 3:**
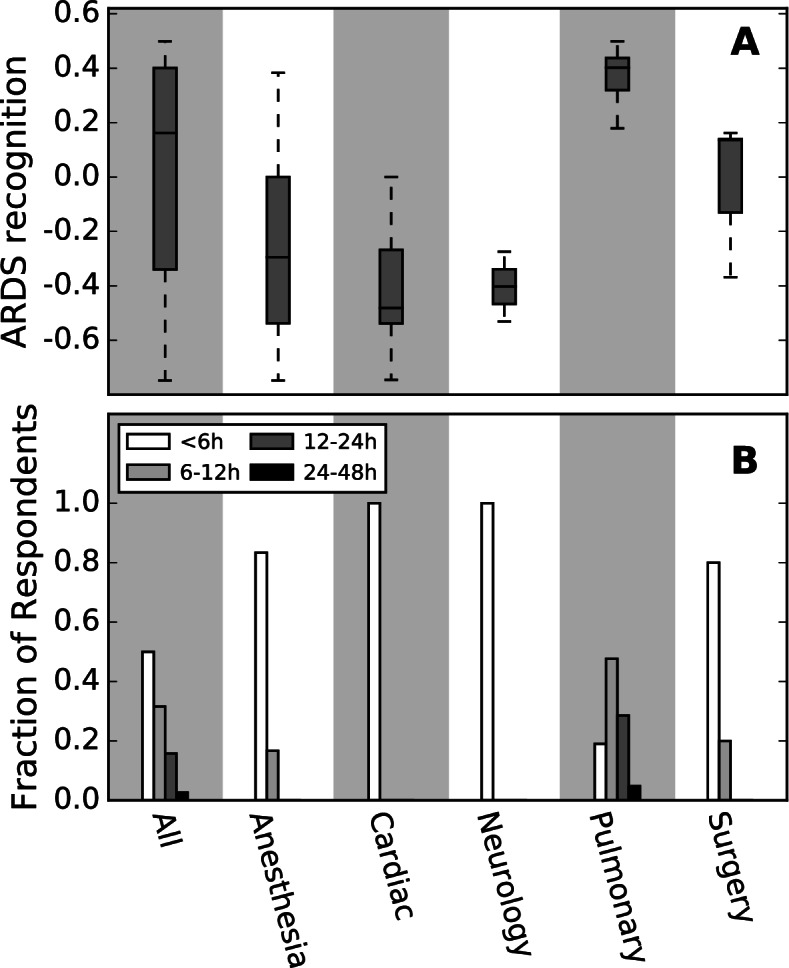
Comparison of ARDS recognition and reported ARDS data wait times between specialties. A) Pulmonary and critical care medicine physicians (PCCM) recognize more ARDS patients than their non-PCCM colleagues. B) PCCM physicians report longer times (hours) to receipt of all data necessary to diagnose ARDS than non-PCCM physicians.

**Fig. 4:**
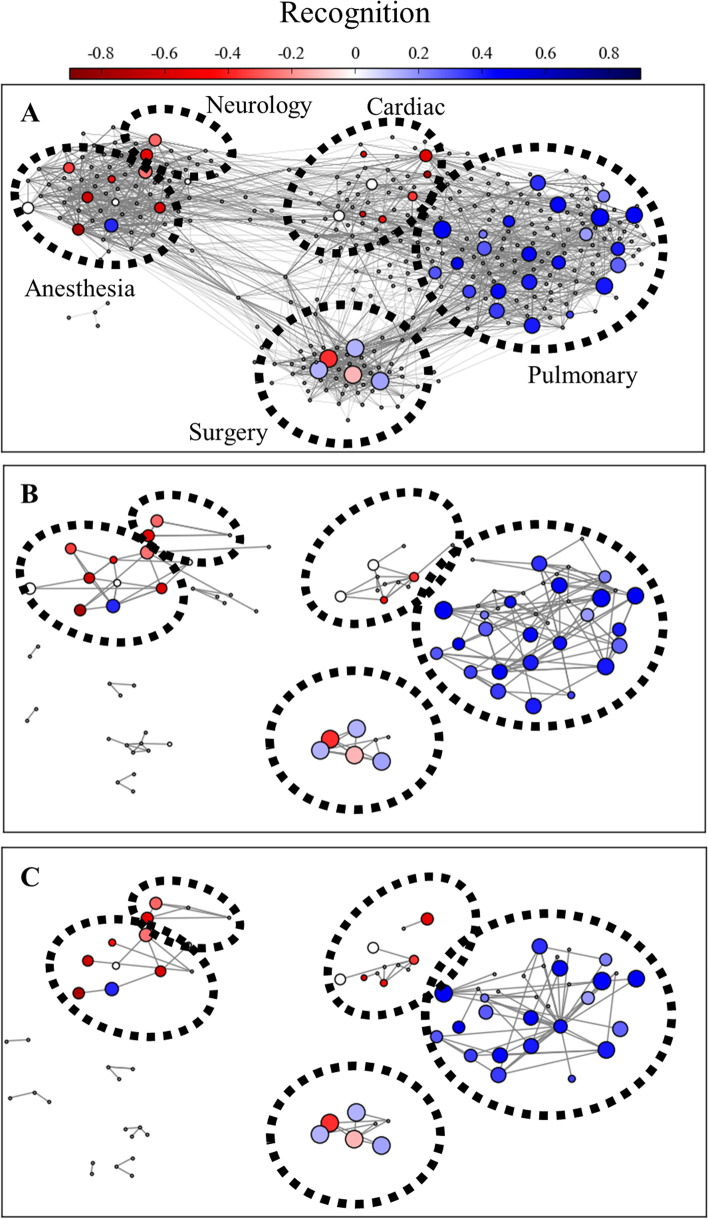
Interaction networks for physicians. Formal interaction networks (A) are based on shared ICU patient care events as determined by attending physician notes. Friendship (B) and opinion-leader (C) networks are built from critical care physicians’ survey responses in which they named colleagues who were considered friends or innovators, respectively. Each circle indicates an individual physician. Marker position is kept constant across network diagrams. Size of marker represents number of ARDS patients cared for by the physician. Color of marker indicates recognition performance (colorbar).

Pulmonary and critical care (PCCM) training showed a significant association with higher recognition (β = 0.63, 95% confidence interval 0.46–0.80, *p* < 7 × 10^− 5^, Fig. 3A). PCCM training was not correlated with a higher number of ARDS patients; those with surgical critical care training cared for the most ARDS patients in our cohort. The patient contact, friendship, and innovation networks are shown in Fig. 4. No positional metric for any network showed any association with recognition. None of the individual survey questions showed a significant association between specific answers and physician recognition.

Since PCCM training was associated with increased recognition, physicians were split into two groups – those with PCCM training and all others – and their survey responses were evaluated for group differences. The only question that showed a statistically significant difference between PCCM and non-PCCM physicians was: “How long does it usually take from the time a patient clinically develops ARDS to the time you receive all the information needed to make a diagnosis of ARDS?” (*p* < 5 × 10^− 4^). Answer options were: < 6 h, 6–12 h, 12–24 h, 24–48 h, and > 48 h. PCCM physicians reported a longer time to ARDS diagnosis as compared to physicians on other teams (6–12 h vs. < 6 h, Fig. 3B). There were no physicians outside the PCCM team that reported ARDS diagnosis times longer than 12 h, with the majority of the non-PCCM physicians reporting < 6 h to ARDS diagnosis (Fig. 3B). The survey included additional systems-specific questions regarding the timing of individual data points (time to lab results, time to chest x-ray results, etc) as well as team-based communication questions, all of which showed no difference in the answers between PCCM physicians and non-PCCM physicians.

##### Study limitations

There are potential limitations to our study that may reduce its generalizability. First, our data was extracted from a single large academic hospital located in a major urban area; the other three centers were smaller, community-based hospitals where data was not collected over the full course of a patient’s stay. Our specific results may not generalize to clinician working at hospitals that differ in one or more of those characteristics. However, we believe that our approach would still be valid for other institutions since it accounts for both patient demographics and institutional setting.

Second, the majority of the networks were built using self-reported interactions, which are potentially biased by subjective reporting. However, the results did not change between directed and undirected networks or when we used the patient contact network for physicians, reducing the potential impact of this bias.

Third, we only built four different network types, which may, or may not, represent the communication network by which physicians would seek advice on ARDS recognition and/or LTVV implementation [46]. It is possible that our lack of association between ARDS recognition and any social network position metric is not a true lack of association, but instead a result of our inability to capture the correct network.

Finally, we did not ask survey respondents to rank barriers with respect to each other, so it is possible that our results concerning the time to obtain all information for an ARDS diagnosis are capturing a heterogenous experience under a single answer. This effect is mitigated by the fact that the question with differences between physician groups actually uses specific time frames, asking the respondents to quantify the delay in receiving the data needed to diagnose ARDS and that no differences were detected when looking at issues with obtaining particular types of data.

## Discussion

Increasing the timely adoption of evidence-based practices is a challenge faced by many industries employing highly-educated professionals such as medicine. In clinical medicine, this challenge is further complicated by the fact that adoption of evidenced-based practices is the two-step process of diagnosis followed by treatment. While missed or delayed diagnoses are important for quality of patient care, these incidents also have an impact on our ability to evaluate the adoption of evidence-based practices. A missed diagnosis is not the same as lack of knowledge about the innovation, or as resistance or refusal to use an evidence-based therapy. It, thus, requires a distinct intervention strategy.

Furthermore, the difficulty of diagnosing a specific disease is not the same patient to patient, with some cases presenting more clearly than others. This two-step adoption process combined with variable diagnosis difficulty makes it incredibly hard to compare the use of evidence-based practices between two physicians (or the same physician over time). In this work, we demonstrate a novel disease recognition metric that accounts for variability in patient disease severity and then use this metric to explore the relationships between acute respiratory distress syndrome (ARDS) recognition and ICU physician characteristics (demographics, social network position, and survey results).

We found that physician training background was strongly associated with ARDS recognition, specifically those physicians trained in pulmonary and critical care medicine (PCCM) recognized ARDS most often. While ARDS is a critical care syndrome, it is primarily a pulmonary condition, and thus it is plausible that pulmonary specialization primes physicians to recognize ARDS. Supporting the hypothesis that training background is a driving factor, we found that those with cardiology training, which does not include critical care certification, comprised the lowest performing group. While the pulmonary and critical care physicians had the most ARDS patient physician pairings, the second highest performing specialty (surgical critical care) had the highest number of ARDS patient contact hours. These results suggest that priming may have an effect on physician ARDS recognition.

Our analyses also uncovered that pulmonary/critical care physicians were more likely to report waiting longer times for receipt of all data necessary for ARDS recognition. PCCM physicians were the only physicians to report any delay greater than 12 h, with 88% of non-PCCM physicians reporting the best possible answer of < 6 h. This finding can be rationalized by the fact that pulmonary/critical care physicians were the top performers: clinicians who are actively engaging with a process, such as ARDS diagnosis, are both more likely to perform well and to be more aware of problems with the recognition process, than clinicians who are less engaged.

Finally, our study found that, once we accounted for the effect of training background, an individual physician’s position within the social or professional networks is not associated with their ability to recognize ARDS. These results hold across all types of connections (advice-seeking, friendship, innovation, patient contact). While a physician’s network position was not associated ARDS recognition, physician communities were primarily composed of a single training background, with very little contact between different critical care specialties. This finding is consistent with prior literature that shows that physicians tend to form tightly knit communities that may calcify knowledge and practices [33, 47–49].

These findings have important implications for the design of interventions and for their implementation. It is a common implementation strategy to survey clinicians about perceived barriers and then design an intervention focused on addressing the most commonly reported barrier [50]. Our findings provide evidence that varying levels of disease recognition and clinician engagement with an innovation should be accounted for when evaluating barriers to adoption. Those physicians whose primary barrier to adoption is disease recognition may provide unintentional false negatives to important survey items. Indeed, our initial survey study showed few perceived barriers to ARDS diagnosis and no consistent correlation between reported barriers or attitudes and evidence-based practice use [26].

## Conclusions

Looking at our results collectively, we found compelling evidence for the existence of two distinct ICU physician populations at a single hospital. Members of these two populations face different challenges in recognizing ARDS. Pulmonary/critical care physicians recognize ARDS more consistently than their peers in other specialties and express concern about accessing the information needed to make this diagnosis. Non-pulmonary/critical care physicians are not able to recognize ARDS consistently and report no barriers in the diagnosis process. These differences suggest that an effective intervention strategy will have to involve distinct approaches for each population.

While our work provides actionable insights for improving ARDS recognition – and subsequent LTVV utilization – a major strength of our methodology is that it accounts for the complexity of the patient population, while remaining agnostic with regard to the specific condition. With this flexibility, our methods can be readily adapted to assess recognition of other complex disease states that have variable presentations, such as mental health disorders. This method allows for the differentiation between which patient-physician interactions constitute adoption opportunities and which interactions are simply disease under-recognition, addressing an important need for effective implementation science in the clinical medicine setting.

## Supplementary Information

**Additional file 1.**

## Data Availability

A limited de-identified dataset representing the final cleaned data analyzed during the current study is available in the public repository managed by Northwestern University: 10.21985/n2-snqm-tk72. Requests for the full data set including dates of admission, intubation, mortality, and full ventilator data will require approval of the Northwestern Institutional Review Board and should be submitted to the corresponding author (amaral@northwestern.edu). Full data will be made available for researchers who meet the criteria for access to confidential data as set forth by the Northwestern Institutional Review Board (https://irb.northwestern.edu/).

## Abbreviations

EBP: Evidence-based practice
LTVV: Low tidal volume ventilation
ARDS: Acute respiratory distress syndrome
ICU: Intensive care unit
PBW: Predicted body weight
P_a_O_2_/F_I_O_2_: Arterial partial pressure of oxygen / fraction of inspired oxygen
OLS: Ordinary least squares
PCCM: Pulmonary and critical care medicine
