# Supplementary material for: The first step is recognizing there is a problem: a methodology for adjusting for variability in disease severity when estimating clinician performance
Source: BMC Med Res Methodol. 2022 Mar 16;22:69. doi: 10.1186/s12874-022-01543-7 (PMC8924737; doi:10.1186/s12874-022-01543-7)
Supplement: Supplementary file 1 — Additional file 1. [file 12874_2022_1543_MOESM1_ESM.zip › Additional FileR3.docx]

**The first step is recognizing there is a problem: a methodology for adjusting disease state recognition for upstream patient presentation variability effects**

**Supplemental Methods**

Probability Density Function Distribution Generation

Steps of the simulation:

- - - 1. Assign each patient a probability of recognition based on their severity
         1. Mild: 0.22
         2. Moderate: 0.34
         3. Severe: 0.67
      2. For each patient, use a random number generator to produce a real number between 0 and 1.
      3. Assign each patient a recognition status by comparing the randomly generated number and the patient’s assigned probability.
         1. If the real number > probability of recognition: assign the patient as **not recognized**
         2. If the real number <= the probability of recognition: assign the patient as recognized.
      4. Sum the total number of patients recognized and store the result.
      5. Repeat steps 2-4 until 1000 iterations have been completed.
      6. Use the 1000 iterations to plot a normalized cumulative probability density function distribution.

Network Construction

We built two major classes of networks: formal and informal. The formal network represents the documented flow of patient care within the hospital as extracted from the electronic health record. A formal network was constructed using only attending physicians as nodes, not limited to critical care. Two physicians were connected if both wrote a note on the same patient’s chart on the same day. The purpose of the formal network was to evaluate an interpersonal interaction network that was not voluntary, and therefore less prone to issues of homophily (i.e., the tendency to seek out those similar to oneself).

We built three different types of informal networks using the survey results reported in (26): advice-seeking, friendship, and traditional opinion leader. Physicians were connected using the answers to four questions in the survey (26):

1. Please write down the names of up to five critical care physicians with whom you work in your ICU whose input you regularly seek to help you make good clinical decisions based on the best available evidence.
2. Please write down the names of up to five critical care physicians with whom you work in your ICU who regularly seek your input to help them make good clinical decisions based on the best available evidence.
3. Please write down the names of up to five critical care physicians with whom you work in your ICU who you consider to be your friends.
4. Please write down the names of up to three critical care physicians with whom you work in your ICU who you think tend to be the first to use new therapies or diagnostic tests.

We labeled the networks built using the answers to questions 1 and 2 as advice-seeking networks, the networks built using the answers to question 3 as friendship networks, and the networks built using the answers to question 4 as innovation networks. Any of these three network types could potentially represent an opinion-leader network (46). We built directed and undirected versions of all informal networks. Questions 1 and 2 also had a frequency component, which was used to build weighted and unweighted versions of the advice-seeking networks. Due to the phrasing of the questions, only within-ICU ties were included informal networks.

**Supp Table 1: Data Availability.**

| **Data element** | **N** | **%** |
| --- | --- | --- |
| Total participants | 92 |  |
| *Demographics* |  |  |
| ICU | 89 | 97 |
| Age | 56 | 61 |
| Sex | 57 | 62 |
| Training year | 55 | 60 |
| *Performance* |  |  |
| ARDS recognition (R) | 48 | 52 |
| Formal Network nodes | 52 | 57 |
| *Survey* |  |  |
| Survey responders | 74 | 80 |
| Reported time between receipt of information and ARDS diagnosis | 68 | 74 |
| Professional network nodes | 88 | 96 |
| Question 1 Answers |  |  |
| Named 1 other physician | 59 | 64 |
| Named 2 other physicians | 55 | 60 |
| Named 3 other physicians | 46 | 50 |
| Named 4 other physicians | 40 | 43 |
| Named 5 other physicians | 26 | 28 |
| Question 2 Answers |  |  |
| Named 1 other physician | 47 | 51 |
| Named 2 other physicians | 39 | 42 |
| Named 3 other physicians | 29 | 31 |
| Named 4 other physicians | 23 | 25 |
| Named 5 other physicians | 18 | 20 |
| Friendship network nodes | 75 |  |
| Question 3 Answers |  |  |
| Named 1 other physician | 49 | 53 |
| Named 2 other physicians | 46 | 50 |
| Named 3 other physicians | 41 | 45 |
| Named 4 other physicians | 35 | 38 |
| Named 5 other physicians | 23 | 25 |
| Opinion Leader network nodes | 65 |  |
| Question 4 Answers |  |  |
| Named 1 other physician | 51 | 55 |
| Named 2 other physicians | 32 | 35 |
| Named 3 other physicians | 19 | 21 |

**Supp Table 2: Node characteristic descriptions.**

| Node Characteristic | Description | Calculation |
| --- | --- | --- |
| Betweenness  (also called ‘brokerage’) | Node placement on paths between other nodes | $x_{i}=\frac{1}{n^{2}}\sum_{st} \frac{n_{st}^{i}}{g_{st}}$  x_i_: betweenness of node i  n: number of nodes in network  $n_{st}^{i}$: number of geodesic paths from s to t that pass through i.  g_st_: number of geodesic paths between s and t |
| Closeness | Distance from all other nodes in the network | $x_{i}=\frac{n-1}{\sum_{st} g_{st}}$  x_i_: closeness of node i  n: number of nodes in network  g_st_: number of geodesic paths between s and t |
| Degree | Number of connections to other nodes. Can be calculated as in and out degree for directed networks. | $x_{i}=\sum_{j} A_{ij}$  x_i_: degree of node i  A_ij_: adjacency matrix of node i |
| Katz Centrality | Measure of centrality that includes not only a node’s degree, but the degree of its immediate neighbors as well. | $x_{i}=\alpha\sum_{j} A_{ij}x_{j}+\beta$  x_i_: Katz centrality of node i  A_ij_: adjacency matrix of node i  α, β: positive weighting constants |
| K-shell embeddedness | Measures the hierarchy of a node within a network. K-shell embeddedness is the maximal subgraph of the network having minimal degree of at least k, but not k+1 | Process:  - prune all nodes with degree ≤ k  - in new graph, prune all nodes with degree ≤ k  - iteratively prune until no nodes exist with degree ≤ k  - all nodes pruned have a k-shell embeddedness of k. |
| Community | A group of nodes that are more connected than would be expected by random chance. | Maximization of modularity as defined by:  $M\equiv\sum_{s=1}^{N_{M}} \left[ \frac{l_{s}}{L}-(\frac{d_{s}}{2L})^{2} \right]$  N_M_: number of communities  L: number of links in network  l_s_: number of links in community s  d_s_: sum of degrees of nodes in community s |
| Participation | Measure of connections node has to communities other than its own | $P_{i}=1-\sum_{s=1}^{N_{M}} (\frac{k_{is}}{k_{i}})^{2}$  P_i_: participation of node i  N_M_: number of communities  k_is_: links to node i in community s  k_i_: total degree of node i |
| Role* (42) | There are 7 distinct roles depending on the location of a node within the participation/in-community degree z-score space. They include: ultra-peripheral (R1), peripheral (R2), non-hub connector (R3), non-hub kinless (R4), provincial hub (R5), connector hub (R6), and kinless hub (R7). | 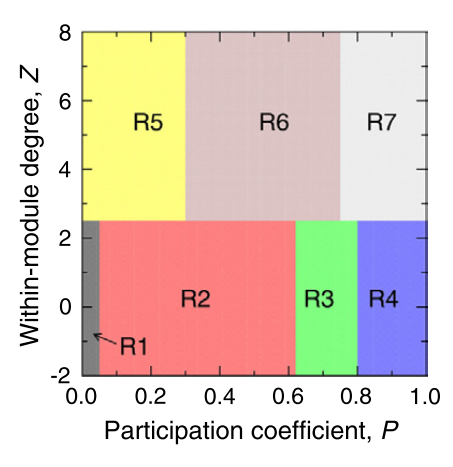 |

* Figure originally published in Guimerà et al. (42) Reproduced here with author permission.

**Supp Table 3: Distribution of ARDS severity by physician group (units of physician-patient pairings).**

|  | All | | Anesthesia/  critical care | | Cardiology | | Neurology/  critical care | | Pulmonary/  critical care | | Surgery/  critical care | |
| --- | --- | --- | --- | --- | --- | --- | --- | --- | --- | --- | --- | --- |
| ARDS severity* | N | % | N | % | N | % | N | % | N | % | N | % |
| Non-ARDS | 44 | 7.8 | 5 | 7.5 | 2 | 5.3 | 0 | 0 | 29 | 10.1 | 8 | 5.1 |
| Mild ARDS | 159 | 28.0 | 23 | 34.3 | 7 | 18.4 | 6 | 33.3 | 80 | 27.9 | 43 | 27.4 |
| Moderate ARDS | 247 | 43.6 | 24 | 35.8 | 19 | 50.0 | 8 | 44.4 | 123 | 42.9 | 73 | 46.5 |
| Severe ARDS | 117 | 20.6 | 15 | 22.4 | 10 | 26.3 | 4 | 22.2 | 55 | 19.2 | 33 | 21.0 |
| Total | 567 |  | 67 |  | 38 |  | 18 | 287 |  | 157 |  |  |

* Lowest P_a_O_2_/F_I_O_2_ during physician-patient pairing.

**Supplemental Figure Legend**

**Supp Figure 1: Comparison of ARDS severity according to physician specialty.**

**Supp Figure 2: ARDS recognition metric is robust to variation within a physician’s cared for patient population.**

A physician’s performance on the ARDS recognition task as calculated by our metric was found to be not associated with the following characteristics of their cared-for patient population: PBW [A) mean, B) median, C) proportion within 1 standard deviation (std) of the mean, D) proportion outside 1 std of the mean, E) proportion outside 2 std of the mean], F) proportion of severe ARDS patients, G) total ARDS patients cared for, and H) proportion of patients that died. Each marker represents an individual physician.
