# Supplementary figures and images for: The first step is recognizing there is a problem: a methodology for adjusting for variability in disease severity when estimating clinician performance
Source: BMC Med Res Methodol. 2022 Mar 16;22:69. doi: 10.1186/s12874-022-01543-7 (PMC8924737; doi:10.1186/s12874-022-01543-7)

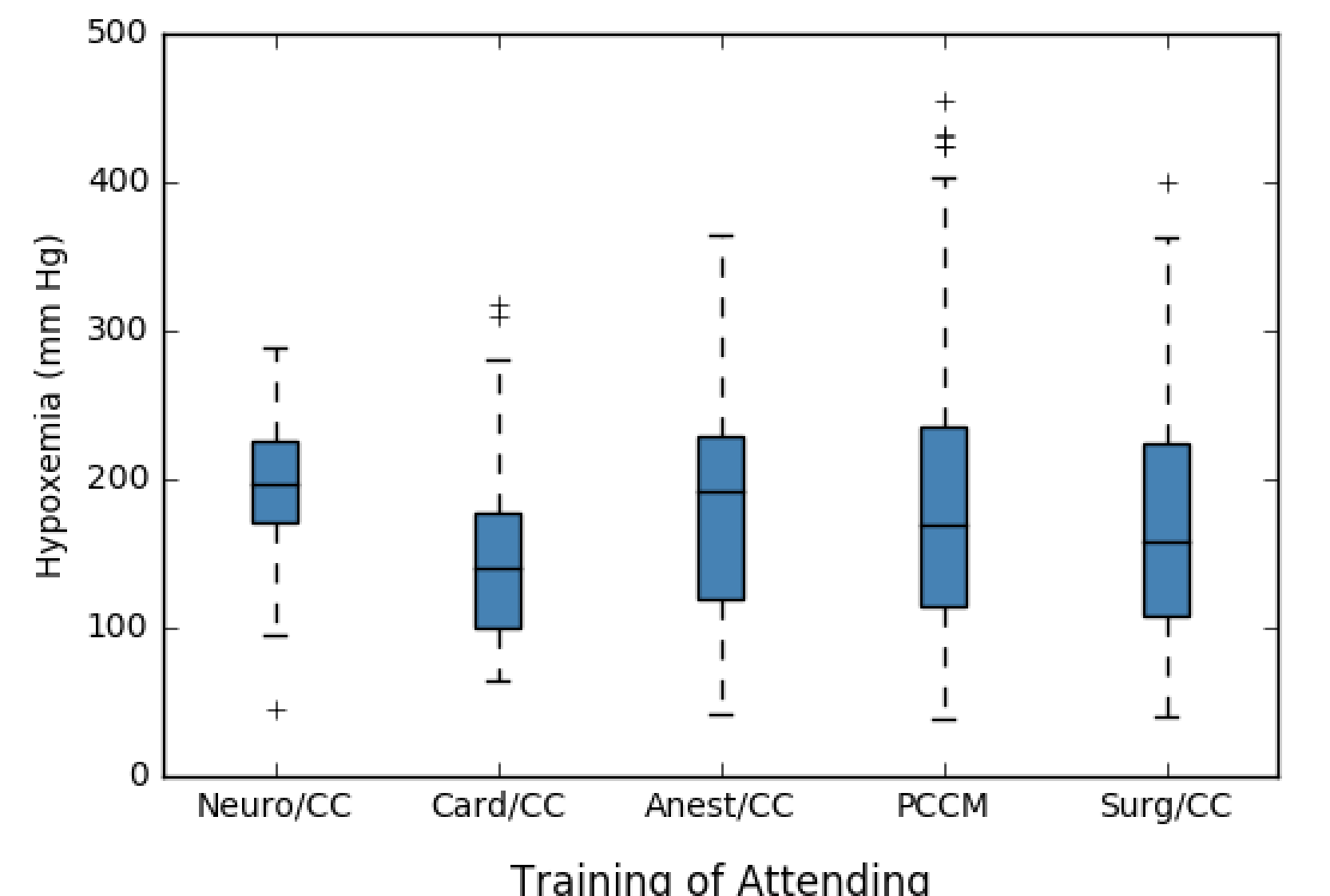

Supplement: Supplementary file 1 — Additional file 1. [file 12874_2022_1543_MOESM1_ESM.zip › Supp Figure 1 FINALR3.tif]

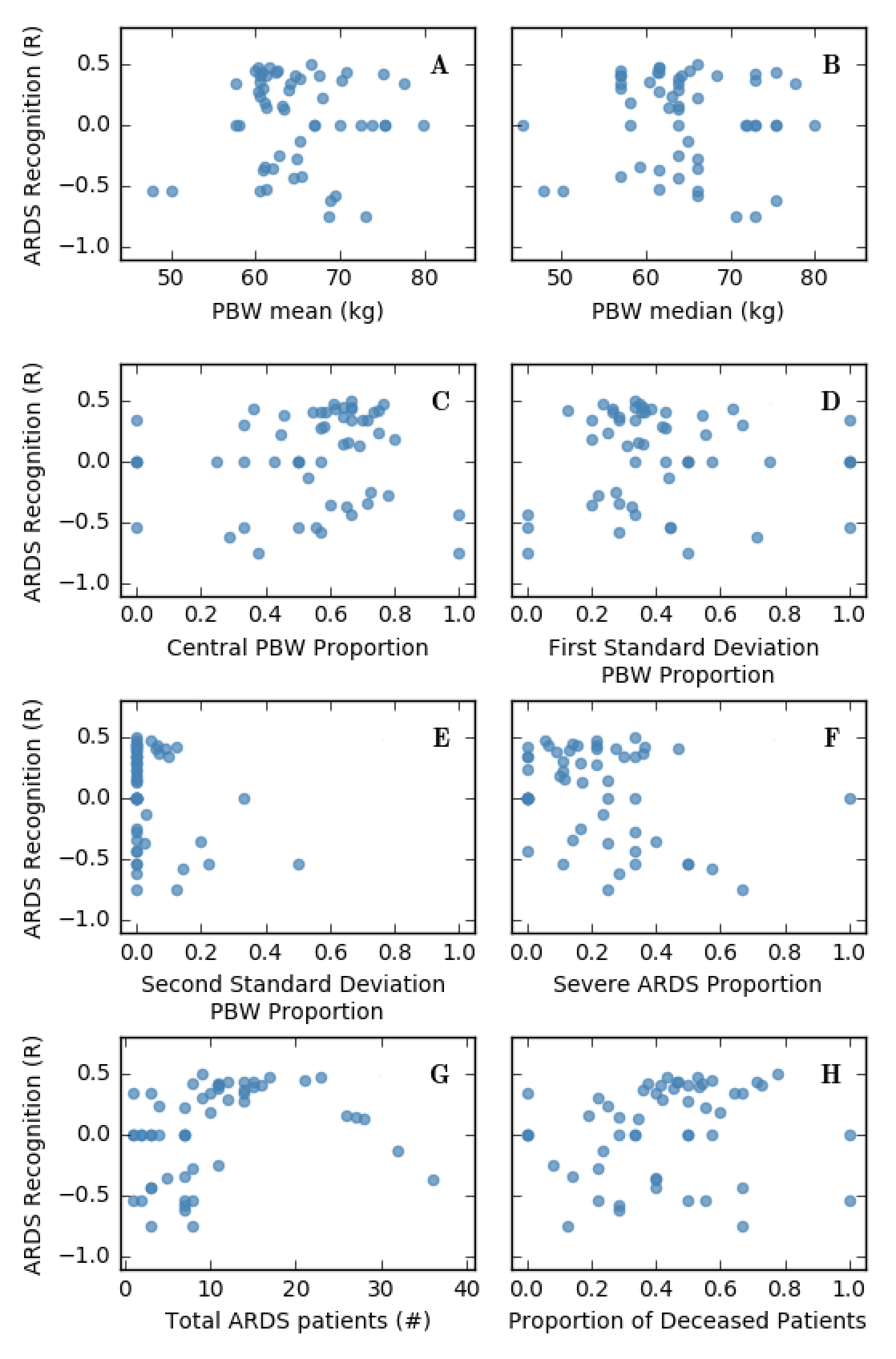

Supplement: Supplementary file 1 — Additional file 1. [file 12874_2022_1543_MOESM1_ESM.zip › Supp Figure 2 FINALR3.tif]
